# Anterior cruciate ligament rupture in a patient with Albers-Schonberg disease

**DOI:** 10.1186/s12891-022-05687-x

**Published:** 2022-07-28

**Authors:** Ke Lu, Biao Cheng, Qin Shi, Xiao-jiao Gao, Chong Li

**Affiliations:** 1 Department of Orthopedics, Affiliated Kunshan Hospital of Jiangsu University, No. 91 West of Qianjin Road, Suzhou, 215300 Jiangsu China; 2 Department of Orthopedics, Shanghai Tenth People’s Hospital, Shanghai, 200072 China; 3 Department of Orthopedics, the First Affiliated Hospital of Soochow University, Orthopedic Institute of Soochow University, Suzhou, 215031 Jiangsu China; 4 Department of Pathology, Affiliated Kunshan Hospital of Jiangsu University, Suzhou, 215300 Jiangsu China

**Keywords:** Osteopetrosis, CLCN7, Mutation, Anterior cruciate ligament, LARS, ADO, Albers-Schonberg disease

## Abstract

**Background:**

Osteopetrosis is an uncommon inherited disease marked with elevated bone density and frequent bone fractures owing to flawed osteoclast activity. Autosomal dominant osteopetrosis type 2 (ADO-2), a benign form of osteopetrosis, is also known as Albers-Schonberg disease.

**Case presentation:**

We report the first successful anterior cruciate ligament (ACL) reconstruction surgery for ACL rupture treatment in a 30-year-old female with ADO-2, who carried a heterozygous missense mutation c.2227C > T (p.Arg743Trp) in exon 23 of the chloride channel 7 (CLCN7) gene. Histopathological analysis of the ruptured ACL sample revealed massive calcium salt deposition in the ligament tissue. A ligament advanced reinforcement system (LARS) artificial ligament was employed in her ACL reconstruction surgery. At her final 16 month’s follow-up, she reported no knee instability symptoms and other complications. The range of motion of the affected knee was good. The side-to-side difference in knee laxity, as evidenced by a KT-1000 arthrometer was 0.9 mm. The Lysholm score improved from 45 before operation to 83 after operation. The Tegner activity score improved from 1 before operation to 4 after operation.

**Conclusions:**

Our findings further confirmed that the newly identified mutated locus (p.Arg743Trp) may lead to acid secretion disorders at different sites (including calcified ACL in our case). In terms of clinical treatment, ligament reconstruction surgery in patients with Albers-Schonberg disease presents a unique challenge to orthopedic surgeons and requires further preparation and time.

## Background

Osteopetrosis (OP) is a clinically and genetically heterogeneous group of diseases characterized by a symmetrical increase in bone density [1]. OP prevalence may range from 1:20,000 to 1:250,000, depending on disease types [1]. It is stratified according to clinical manifestations, genetic inheritance, and discrete signaling pathways. A myriad of clinical manifestations and genetic inheritance are associated with the different forms of OP. Genetic inheritance can be autosomal recessive, autosomal dominant or X-linked. Autosomal recessive osteopetrosis (ARO), an OP malignancy detected in infancy, can impair growth and dramatically increase fracture risk. ARO patients often experience anemia and recurring infections. This is likely due to bone expansion, which narrows the bone marrow space and eventually leads to extramedullary hematopoiesis. Some ARO patients also experience blindness, facial paralysis, and deafness, owing to excessive bone pinching on cranial nerves [1].

Interestingly, patients with autosomal dominant osteopetrosis (ADO), a benign form of OP, are often asymptomatic. ADO onset is usually in late childhood or adolescence and it can be separated in three subcategories: benign type 1 (ADO-1), benign type 2 (ADO-2), and benign type 3 (ADO-3) [2]. Among them, ADO-2, also known as Albers-Schonberg disease, is the most reported form of OP. ADO-2 is manifested by vertebral endplate thickening ‘sandwich vertebrae’, along with excessive cortical, but normal cancellous bone volume, brittle bones, and increased fracture risk late in life [3–5]. Among the most prevalent causes of ADO-2 is an inactivating mutation in the chloride channel 7 (CLCN7) gene, which diminishes osteoclast activity via impaired acidification of the osteoclast resorption lacunae, thereby abolishing bone mineral degradation [6].

Tears of the anterior cruciate ligament (ACL) are among the most frequently studied injuries in orthopedic literature. A population-based cohort study carried out in Minnesota, United States, found that the annual incidence of new ACL injuries, adjusted for sex and age, was 68.6 per 100,000 people [7]. Although some papers reported orthopaedical surgery as a likely treatment for ADO-2-related fractures [8, 9], the treatment of anterior cruciate ligament (ACL) rupture in ADO-2 patients is rarely reported. In addition, there are limited familial genetic analyses as well. Here, we introduce a case report of an ADO-2 patient, with heterozygous gene mutation in CLCN7 gene, who received ACL reconstruction surgery in China.

## Case presentation

A 30-year-old woman sought treatment at our joint surgery department for pain and instability of the right knee joint, after suffering from injury. She experienced a valgus semi-extension injury to her knee while driving an electric motorcycle that was hit by a car. MRI examination revealed complete ACL rupture.

**Fig. 1:**
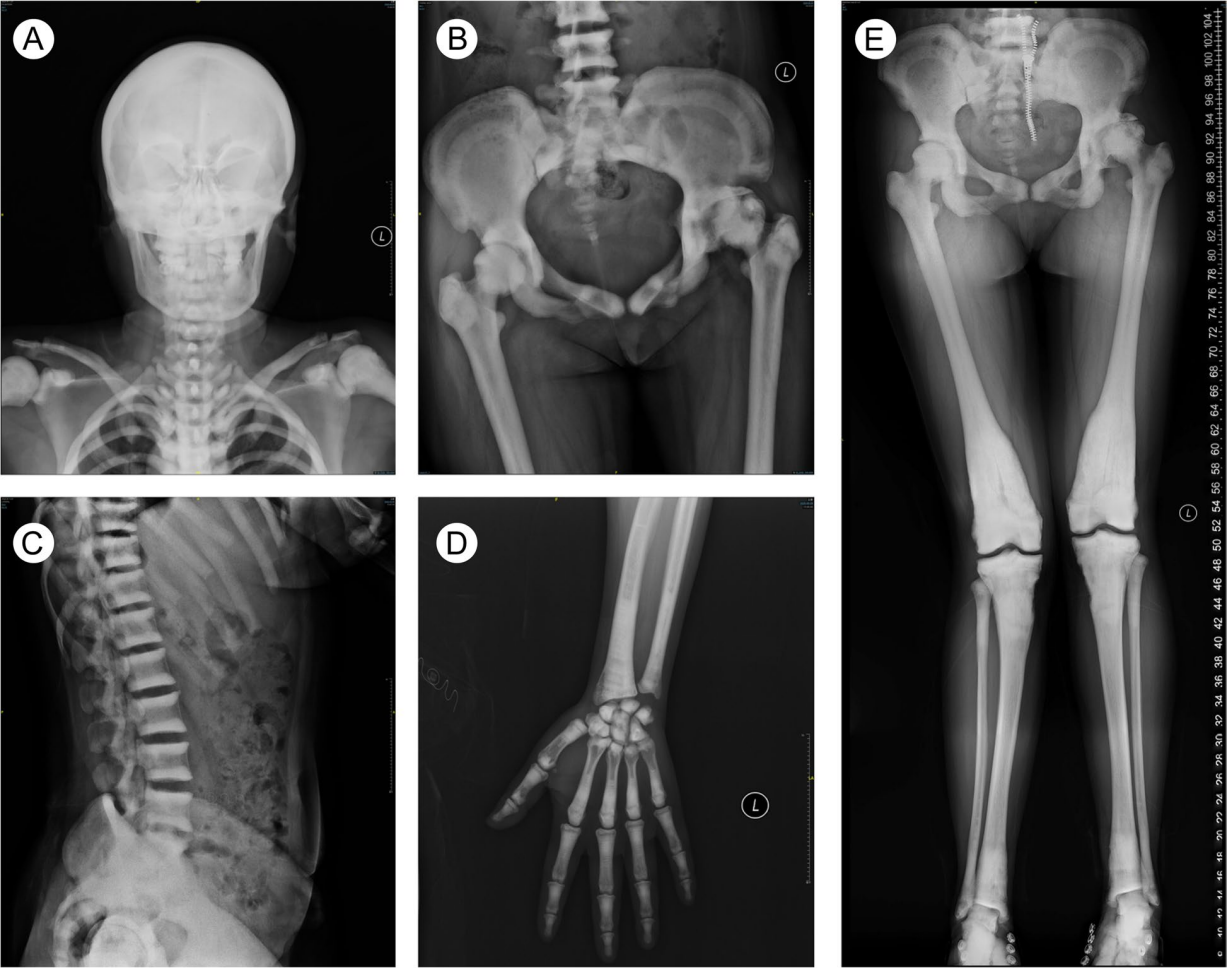
X-ray evaluation of an autosomal dominant osteopetrosis type 2 (ADO-2) phenotype. **A** AP skull radiograph showing generalized increase in bone density. **B** Sclerosis in the iliac wings, acetabuli and femoral heads. **C** Lateral thoracic and lumbar spine radiograph with dense sclerotic bands at the vertebral body endplates (‘sandwich vertebrae’). **D** AP phalanges radiograph with endobone (‘bone-in-bone’) appearance. **E** A full-length standing anteroposterior radiograph of both lower limbs showing diffused bone sclerosis, ‘Erlenmeyer flask’ deformity, and ‘bone-in-bone’ appearance. AP = anterior–posterior

The patient had a history of OP and was diagnosed with the disease at the time of her first fracture at the age of 13. Radiographs showed diffused sclerosis in the skull, vertebrae, pelvis, and appendicular bones. Knee X-ray revealed typical bone modelling defects (‘Erlenmeyer flask’ deformity). There was obvious ‘bone-in-bone’ morphology at the femur heads, vertebrae, and phalanges. In addition, focal sclerosis was detected in the skull base, pelvis, and vertebral end plates (‘sandwich vertebrae’) (Fig. 1). Bone mineral densitometry (BMD) of the antero-posterior lumbar spine vertebrae, L1-L4, was 2·381 g/cm^2^ (Z-score = 12.1), as evidenced by dual-energy X-ray absorptiometry. The BMD of the left femoral neck was 1.863 g/cm^2^ (Z-score = 9.2). Blood chemistry exhibited elevated levels of N-terminal propeptide of type I collagen (142 ng/mL [reference range 16–55 ng/mL]).

**Fig. 2:**
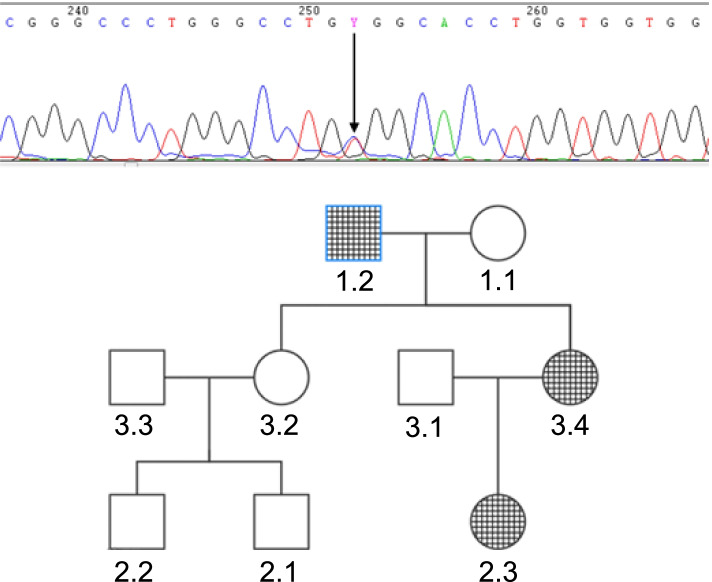
A heterozygous missense mutation identified in a proband, her father, and her daughter. A heterozygous C to T transition (arrow) is shown at position 2227 in exon 23 of the chloride channel 7 gene, replacing an arginine with a tryptophan, with substitution at codon position 743 (NM_001114331.2 [CLCN7]: c.2227C > T, p.Arg743Trp). 3.4 = proband, 1.2 = father, 2.3 = daughter

To evaluate the genetics of OP, a targeted gene panel was sequenced to investigate the presence of pathogenic variants of multiple OP-related genes. With informed consent, 3-mL blood samples were collected from the patient, her parents, 6-year-old daughter, older sister, and two nephews. Based on our analysis, the same heterozygous missense mutation c.2227C > T (p.Arg743Trp) was observed in exon 23 of the CLCN7 gene in the proband, her father, and her daughter, in whom the mutated locus was first reported (Fig. 2). The family was assumed to suffer from ADO-2. Nevertheless, the father and daughter had no OP-related complaints and their other relevant tests appeared normal.

**Fig. 3:**
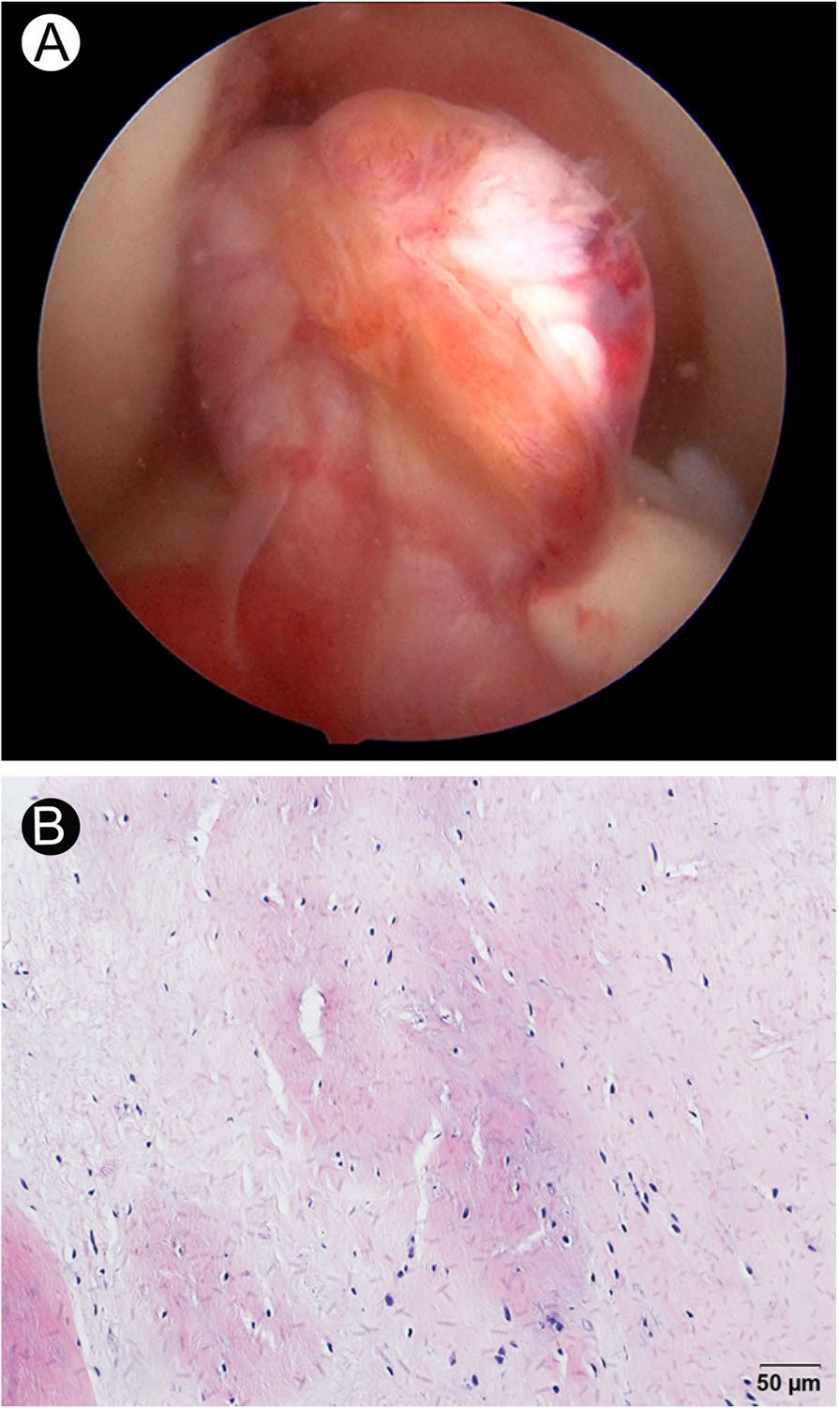
Anterior cruciate ligament rupture in an ADO-2 patient. **A** Arthroscopy showing complete ACL rupture. **B** Histopathological analysis of the ruptured ACL sample depicting calcium salt deposition (haematoxylin and eosin stain). Original magnification × 200. ADO-2 = autosomal dominant osteopetrosis type 2. ACL = anterior cruciate ligament

At 8 weeks after the injury, the patient underwent ACL reconstruction surgery, using a ligament advanced reinforcement system (LARS) (Surgical Implant and Devices, Arc-sur-Tille, France) artificial ligament (AC 100 [8 mm diameter, 100 free fibers]), following earlier reported isometric surgical principles [10]. Due to the significant increase in bone density, the guide pin, and hollow drill bits were not able to penetrate the tough bone. Hence, a bone tunnel was established with the direct use of a 7.5 mm diameter solid drill bit. The 9.0 mm diameter titanium interference screws were also extremely difficult to screw in. In all, the operation lasted 130 min and was successfully completed. Histopathological analysis of the ruptured ACL sample, obtained during the operation, revealed massive deposition of calcium salts in the ligament tissue (Fig. 3). The patient was discharged from the hospital 3 days after the operation. She underwent a standardized rapid rehabilitation program, as described previously [11].

**Fig. 4:**
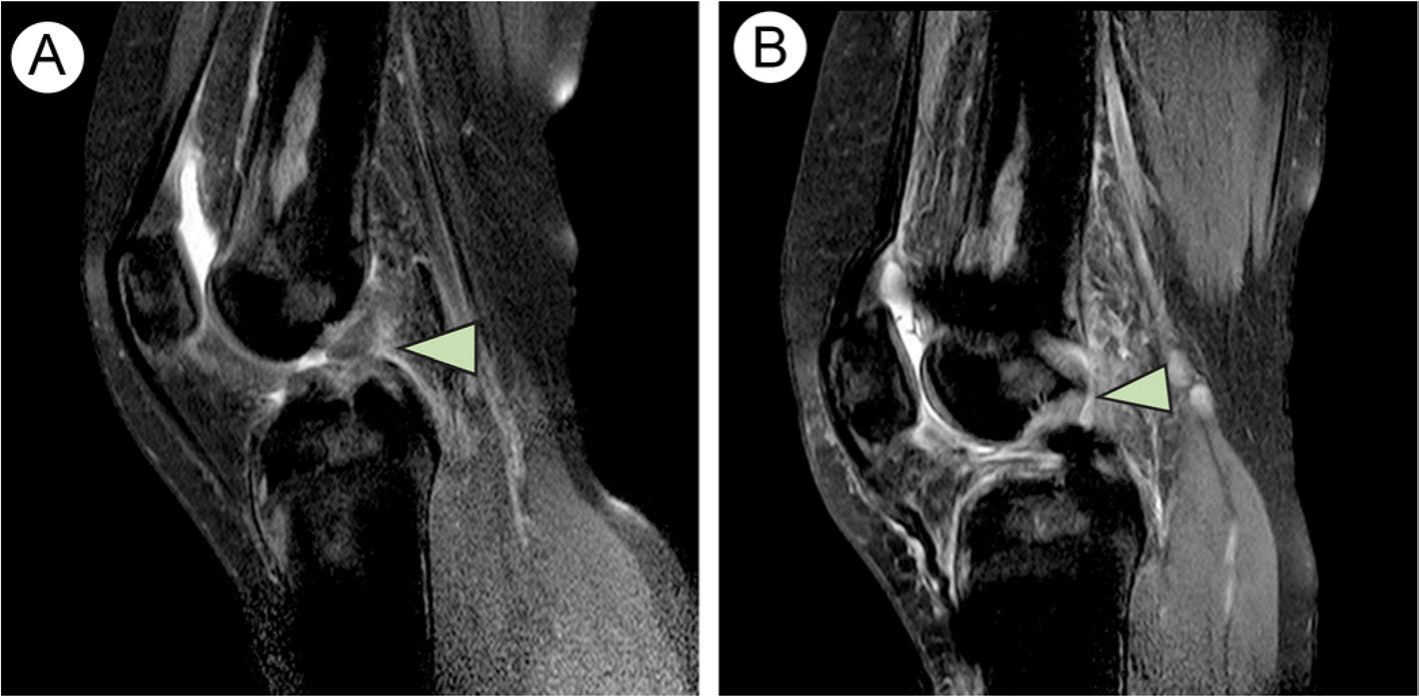
Preoperative and postoperative MRI findings. **A** Preoperative T2-weighted MRI illustrating complete ACL rupture (arrow). **B** Postoperative T2-weighted MRI illustrating LARS ligament after ACL reconstruction (arrow). LARS = Ligament Augmentation and Reconstruction System

Postoperative 1 month’s T2-weighted MRI illustrated the LARS ligament was intact after ACL reconstruction (Fig. 4). Until her final 16 month’s follow-up, she reported no knee instability symptoms and other complications. Clinical assessment involved both passive and active range of motion (ROM) and the KT-1000 arthrometer (MED metric, San Diego, CA, USA), which assessed the side-to-side difference between the affected and nonaffected knee. The patient also filled out validated patient-reported outcome measure surveys including the Lysholm knee scoring scale and Tegner activity score [12]. The flexion angle of the affected knee was 115° and that of nonaffected knee was 125°. The extension angle of both side of the knee was 0°. The side-to-side difference in knee laxity, as evidenced by a KT-1000 arthrometer was 0.9 mm. The Lysholm score improved from 45 before operation to 83 after operation. The Tegner activity score improved from 1 before operation to 4 after operation.

## Discussion and conclusions

This is the first report of an ADO-2 patient, with p.Arg743Trp mutation in the CLCN7 gene, undergoing ACL reconstruction surgery described in the English literature. Histopathological analysis of the ruptured ACL sample revealed massive deposition of calcium salts in the ligament tissue. Her surgical treatment of ACL rupture represented a unique challenge to the orthopedic surgeon due to her ADO-2 diagnosis, but short-term follow-up results showed that the surgery was successful in that the reconstructed ACL was stable and the knee had a good range of motion.

Earlier reports established that ADO-2 is caused by a mutation in chromosome 16p13.3. In later research, this mutation was found to be in the CLCN7 gene [4]. This gene carries the genetic code for the chloride channel 7 protein subunit (ClC-7). This subunit comprises of 803 amino acids and is responsible for maintaining efficiency in proton pumping in the osteoclast ruffled membrane [6]. Osteoclasts resorb bone by secreting proteases and hydrochloric acid, which work together to dissolve the calcified bone matrix [13]. Active proton transport, regulated by osteoclast-specific V-ATPase, is essential to this process as it acidifies the resorption compartment via chloride secretion modulated by the chloride-proton antiporter ClC-7 [14]. Hence, individuals with CLCN7 mutation often experience diminished bone resorption and eventually develop OP [15]. The CLCN7 mutations associated with ADO-2 are highly varied, with over 200 mutations identified so far [16]. In this report, we introduce a new heterozygous missense mutation c.2227C > T (p.Arg743Trp) in the CLCN7 gene. Interestingly, the same mutated locus was previously reported in the ATP6V0A4 gene in a recessive distal renal tubular acidosis (dRTA) patient [17]. ATP6V0A4 genes encode a4 subunits of the V-ATPase. The V-ATPase is located in the acid secretory alpha intercalated cells of the renal cortical and medullary collecting ducts as well as the cochlear endolymphatic sac epithelial cells. Mutations in these genes dysregulate V-ATPase proton-secreting activity and induce the autosomal recessive form of dRTA, which is related to sensorineural hearing loss [18]. In our case, the proband, her father, and daughter were asymptomatic in terms of kidney function and hearing. We suspected that there are modifying factors that determine whether a given mutation carrier becomes symptomatic. Indeed, our findings further confirmed that the newly identified mutated locus (p.Arg743Trp) may lead to acid secretion disorders at different sites (including calcified ACL in our case), resulting in different clinical symptoms. Thus, our analysis of the novel mutated locus may be a good indicator for future examinations and molecular diagnostic of diseases caused by mutations in the CLCN7 gene.

On the clinical treatment side, relative to normal patients, orthopedic surgery takes longer in ADO-2 patients, and the surgeon faces extra challenges like drill bit breakage and excessive blood loss [8]. Moreover, lack of knowledge about OP, along with insufficient preoperative examination can result in both pre- and postoperative, complications. So, in our case, we performed an extensive preoperative evaluation and preparation, including special surgical tools like solid and sharper bits. Despite this, we still encountered few difficulties during the operation, described as follows: the Kirschner wire could not be used for positioning and interface screws were difficult to screw in.

Considering the poor quality of our patient’s calcified ligament autografts and the potential risk of infections at the donor site, we used artificial LARS ligaments instead. Recently, a study revealed that despite the favorable long-term overall satisfaction of patients, who have undergone ACL reconstruction with the LARS device, the high cumulative reoperation rate was 51% and complication rate was 66% [19]. Thus, the long-term outcome of the OP patient using the LARS ligaments requires further observation. In addition, the autogenous reconstruction with four-strand hamstrings or bone-patellar tendon grafts is a widely accepted treatment after ACL rupture [20], the efficacy of autogenous reconstruction in patients with OP require further examination. Finally, we speculate that allograft tendons are among the donor options that may be considered since the femoral suspension devices of allograft tendons can minimize interface screws usage, thus partially reducing the difficulty of ligament reconstruction surgery in patients with OP.

In conclusion, our findings further confirmed that the newly identified mutated locus (p.Arg743Trp) may lead to acid secretion disorders at different sites (including calcified ACL in our case). In terms of clinical treatment, ligament reconstruction surgery in patients with Albers-Schonberg disease presents a unique challenge to orthopedic surgeons and requires further preparation and time.

## Data Availability

The datasets used and/or analyzed during the current study are available from the corresponding author on reasonable request.

## Abbreviations

ADO-2: Autosomal dominant osteopetrosis type 2
ACL: Anterior cruciate ligament
CLCN7: Chloride channel 7
LARS: Ligament advanced reinforcement system
OP: Osteopetrosis
ARO: Autosomal recessive osteopetrosis
ADO: Autosomal dominant osteopetrosis
BMD: Bone mineral densitometry
ROM: Range of motion
ClC-7: Chloride channel 7 protein subunit
dRTA: Distal renal tubular acidosis
AP: Anterior–posterior

## References

[1] Stoker DJ (2002). Osteopetrosis. Semin Musculoskelet Radiol.

[2] Balemans W, Van Wesenbeeck L, Van Hul W (2005). A clinical and molecular overview of the human osteopetroses. Calcif Tissue Int.

[3] Bénichou OD, Laredo JD, de Vernejoul MC (2000). Type II autosomal dominant osteopetrosis (Albers-Schönberg disease): clinical and radiological manifestations in 42 patients. Bone.

[4] Cleiren E, Bénichou O, Van Hul E, Gram J, Bollerslev J, Singer FR, Beaverson K, Aledo A, Whyte MP, Yoneyama T, deVernejoul MC, Van Hul W (2001). Albers-Schönberg disease (autosomal dominant osteopetrosis, type II) results from mutations in the ClCN7 chloride channel gene. Hum Mol Genet.

[5] Zhang ZL, He JW, Zhang H, Hu WW, Fu WZ, Gu JM, Yu JB, Gao G, Hu YQ, Li M, Liu YJ (2009). Identification of the CLCN7 gene mutations in two Chinese families with autosomal dominant osteopetrosis (type II). J Bone Miner Metab.

[6] Tolar J, Teitelbaum SL, Orchard PJ (2004). Osteopetrosis. N Engl J Med.

[7] Sanders TL, MaraditKremers H, Bryan AJ, Larson DR, Dahm DL, Levy BA, Stuart MJ, Krych AJ (2016). Incidence of Anterior Cruciate Ligament Tears and Reconstruction: A 21-Year Population-Based Study. Am J Sports Med.

[8] Yiğit Ş, Arslan H, Akar MS, Şahin MA (2020). Mid-term outcomes of surgical treatment in fractures in patients with osteopetrosis. Bone Joint J..

[9] Kim J, Park YC, Moon HS, Do WS, Yang KH (2020). Intramedullary nailing for subtrochanteric fracture in autosomal dominant Type II osteopetrosis: Case report of 2 patients. Medicine (Baltimore).

[10] Dericks G (1995). Ligament advanced reinforcementsystem anterior cruciate ligament reconstruction. Operative Techniques in Sports Medicine.

[11] Tulloch SJ, Devitt BM, Porter T, Hartwig T, Klemm H, Hookway S, Norsworthy CJ (2019). Primary ACL reconstruction using the LARS device is associated with a high failure rate at minimum of 6-year follow-up. Knee Surg Sports Traumatol Arthrosc.

[12] Collins NJ, Misra D, Felson DT, Crossley KM, Roos EM (2011). Measures of knee function: International Knee Documentation Committee (IKDC) Subjective Knee Evaluation Form, Knee Injury and Osteoarthritis Outcome Score (KOOS), Knee Injury and Osteoarthritis Outcome Score Physical Function Short Form (KOOS-PS), Knee Outcome Survey Activities of Daily Living Scale (KOS-ADL), Lysholm Knee Scoring Scale, Oxford Knee Score (OKS), Western Ontario and McMaster Universities Osteoarthritis Index (WOMAC), Activity Rating Scale (ARS), and Tegner Activity Score (TAS). Arthritis Care Res..

[13] Teitelbaum SL (2007). Osteoclasts: what do they do and how do they do it?. Am J Pathol.

[14] Henriksen K, Flores C, Thomsen JS, Brüel AM, Thudium CS, Neutzsky-Wulff AV, Langenbach GE, Sims N, Askmyr M, Martin TJ, Everts V, Karsdal MA, Richter J (2011). Dissociation of bone resorption and bone formation in adult mice with a non-functional V-ATPase in osteoclasts leads to increased bone strength. PLoS ONE.

[15] Del Fattore A, Cappariello A, Teti A (2008). Genetics, pathogenesis and complications of osteopetrosis. Bone.

[16] Pangrazio A, Pusch M, Caldana E, Frattini A, Lanino E, Tamhankar PM, Phadke S, Lopez AG, Orchard P, Mihci E, Abinun M, Wright M, Vettenranta K, Bariae I, Melis D, Tezcan I, Baumann C, Locatelli F, Zecca M, Horwitz E, Mansour LS, Van Roij M, Vezzoni P, Villa A, Sobacchi C (2010). Molecular and clinical heterogeneity in CLCN7-dependent osteopetrosis: report of 20 novel mutations. Hum Mutat.

[17] Escobar LI, Simian C, Treard C, Hayek D, Salvador C, Guerra N, Matos M, Medeiros M, Enciso S, Camargo MD, Vargas-Poussou R (2016). Mutations in ATP6V1B1 and ATP6V0A4 genes cause recessive distal renal tubular acidosis in Mexican families. Mol Genet Genomic Med.

[18] Vargas-Poussou R, Houillier P, Le Pottier N, Strompf L, Loirat C, Baudouin V, Macher MA, Déchaux M, Ulinski T, Nobili F, Eckart P, Novo R, Cailliez M, Salomon R, Nivet H, Cochat P, Tack I, Fargeot A, Bouissou F, Kesler GR, Lorotte S, Godefroid N, Layet V, Morin G, Jeunemaître X, Blanchard A (2006). Genetic investigation of autosomal recessive distal renal tubular acidosis: evidence for early sensorineural hearing loss associated with mutations in the ATP6V0A4 gene. J Am Soc Nephrol.

[19] Smolle MA, Fischerauer SF, Zötsch S, Kiegerl AV, Sadoghi P, Gruber G, Leithner A, Bernhardt GA (2022). Long-term outcomes of surgery using the Ligament Advanced Reinforcement System as treatment for anterior cruciate ligament tears. Bone Joint J..

[20] Gao K, Chen S, Wang L, Zhang W, Kang Y, Dong Q, Zhou H, Li L (2010). Anterior cruciate ligament reconstruction with LARS artificial ligament: a multicenter study with 3- to 5-year follow-up. Arthroscopy.

